# 

**Published:** 1950-04

**Authors:** 


### CONTENTS

Page

**Poliomyelitis in Bristol, 1949.** By JAMES MACRAE, M.D., F.R.F.P.S., D.P.H. .. 43
**Epidemic Myalgia—Bornholm Disease.** By N. S. CRAIG, *M.C.* M.D. 50
**Gastric Ulcer with Perforation into the Lesser Sac:** *Report of a case.* By J. A. VERE NICOLL, F.R.C.S. .. 53
**Congenital Atresia of the Oesophagus:** *Reports of three cases.* By RONALD BELSEY, F.R.C.S. .. 54
**Some Recent Advances in Paediatrics.** By WILFRID P. H. SHELDON, M.D., F.R.C.P. .. 56
**Reviews of Books** .. 59
**Meetings of Societies** .. 61
**Local Medical Notes** .. 65

